# Effective Connectivity Analysis of the Brain Network in Drivers during Actual Driving Using Near-Infrared Spectroscopy

**DOI:** 10.3389/fnbeh.2017.00211

**Published:** 2017-10-31

**Authors:** Zhian Liu, Ming Zhang, Gongcheng Xu, Congcong Huo, Qitao Tan, Zengyong Li, Quan Yuan

**Affiliations:** ^1^Key Laboratory of High Efficiency and Clean Mechanical Manufacture, School of Mechanical Engineering, Shandong University, Jinan, China; ^2^Interdisciplinary Division of Biomedical Engineering, Faculty of Engineering, Hong Kong Polytechnic University, Kowloon, Hong Kong; ^3^Beijing Key Laboratory of Rehabilitation Technical Aids for Old-Age Disability, National Research Center for Rehabilitation Technical Aids, Beijing, China; ^4^Key Laboratory of Rehabilitation Aids Technology and System of the Ministry of Civil Affairs, Beijing, China

**Keywords:** near-infrared spectroscopy, effective connectivity, Granger causality, actual driving, cognitive workload

## Abstract

Driving a vehicle is a complex activity that requires high-level brain functions. This study aimed to assess the change in effective connectivity (EC) between the prefrontal cortex (PFC), motor-related areas (MA) and vision-related areas (VA) in the brain network among the resting, simple-driving and car-following states. Twelve young male right-handed adults were recruited to participate in an actual driving experiment. The brain delta [HbO_2_] signals were continuously recorded using functional near infrared spectroscopy (fNIRS) instruments. The conditional Granger causality (GC) analysis, which is a data-driven method that can explore the causal interactions among different brain areas, was performed to evaluate the EC. The results demonstrated that the hemodynamic activity level of the brain increased with an increase in the cognitive workload. The connection strength among PFC, MA and VA increased from the resting state to the simple-driving state, whereas the connection strength relatively decreased during the car-following task. The PFC in EC appeared as the causal target, while the MA and VA appeared as the causal sources. However, l-MA turned into causal targets with the subtask of car-following. These findings indicate that the hemodynamic activity level of the cerebral cortex increases linearly with increasing cognitive workload. The EC of the brain network can be strengthened by a cognitive workload, but also can be weakened by a superfluous cognitive workload such as driving with subtasks.

## Introduction

Driving a vehicle is a complex activity that requires high-level brain functions, such as planning, decision making, visual attention, motor control and high cognitive activity to make fast cognitive decisions in a complex and rapidly changing environment (Derosière et al., 2014). Some individuals may be able to drive under simple conditions, but may be incapable of driving safely when cognitive workloads become heavier, such as following a proceeding car, talking on the phone and performing other subtasks (Uchiyama et al., 2003; Schweizer et al., 2013). Several studies have reported that some brain regions, such as the prefrontal cortex (PFC), motor-related areas (MA), parietal cortex, vision-related areas (VA), thalamus and cerebellum, engage in high-level cognitive activity during car driving with functional near-infrared spectroscopy (fNIRS; Yoshino et al., 2013a,b; Orino et al., 2015). However, little information is known on how the functional networks are affected by the cognitive workload during actual driving. Therefore, it makes sense to analyze driving data in a manner that evaluates the brain activation among regions, which enables us to study how the brain is functionally connected and how these intrinsic networks are modulated by the cognitive workload (Calhoun and Pearlson, 2012).

The brain activation was identified with functional magnetic resonance imaging (fMRI) and their modulation with speed was investigated during simulated driving (Calhoun et al., 2002). The authors found that the signal in frontoparietal regions decreased exponentially with a rate proportional to the driving speed. They also found that increases in the cerebella and occipital areas, presumably related to the complex visuomotor integration, were activated during driving but not associated with the driving speed (Calhoun et al., 2002).

The brain activation among cortex areas was found to differ with familiar or unfamiliar routes. For example, significant activation was found in the middle temporal and occipital cortex and in the cerebellum for the unfamiliar route. A training period and a familiar, monotonous route may lead to a reduction in attention and perception (Mader et al., 2009). Furthermore, the brain activity assessed in terms of regional response and regional interactions in highly trained racing-car drivers was found to differ from that of subjects with an ordinary driving experience (Bernardi et al., 2014).

The brain activation during actual car-driving on the road was demonstrated to be similar to that of simulated driving and visual perception and visuomotor coordination were the main brain functions while driving (Jeong et al., 2006). However, autonomic and emotional responses such as attention and autonomic arousal should be considered using actual driving (Jeong et al., 2006).

fNIRS has been proven to be a reliable method to represent the cortical activities of the brain network during driving (Tsunashima and Yanagisawa, 2009; Yoshino et al., 2013b; Lin and Lin, 2016), which monitors spontaneous hemodynamic oscillations from cortical regions. Compared with other neuroimaging methods such as fMRI, fNIRS requires relatively few physical constraints and is suitable for measuring moving subjects. Moreover, fNIRS exhibits a higher sampling rate (approximately 10 Hz) than fMRI (approximately 1 Hz), implying a better time resolution.

Several studies show that functional connectivity (FC) can be assessed using fNIRS data in drivers (Wang W. et al., 2016; Xu et al., 2017). FC is generally inferred by the correlation between nodal activities on the basis of blood oxygenation level-dependent fMRI or coherence in electro- or magneto- encephalogram signals acquired during task performance or the resting state (Park and Friston, 2013). However, FC does not provide the mechanisms of neuronal coupling. Effective connectivity (EC) is defined as the influence that a node exerts over another under a network model of causal dynamics and is inferred from a model of neuronal integration, which defines the mechanisms of neuronal coupling (Friston, 2011).

EC is usually computed using several methods, including dynamic causal modeling (DCM; Friston et al., 2003), Granger causality (GC; Granger, 1969), and structural equation modeling (SEM; Anderson and Gerbing, 1988). The basic idea of GC was proposed by Wiener and was then successively formalized by Granger using an autoregressive model (Granger, 1969; Bressler and Seth, 2011). Subsequently, Geweke introduced the conditional GC (CGC) algorithm to extend GC to a conditional case (Geweke, 1982). Recently, GC has been used to assess the directionality of neuronal interactions in both the frequency and the time domain (Im et al., 2010; Sitaram et al., 2014; Anwar et al., 2016; Cai et al., 2016). Compared with GC, SEM becomes unstable when the data is presented as a time series, while DCM always requires the selection of a prior model in advance, which may prove to be a very complex process because of the presence of multiple variables. The EC networks among overlapping core regions recruited by motor execution and motor imagery were explored by using CGC and on fMRI data. It was demonstrated that more circuits of EC among the selected seed regions were activated during right-hand performance than during left-hand performance (Gao et al., 2011).

This study hypothesizes that driving as a complex activity would activate a brain interaction among different brain areas and strengthen the EC of the brain network compared to the resting state. Given that PFC significantly contributes to cognitive behavior (Miller and Cohen, 2001), MA is highly associated with sensation and movement control (Peterka, 2002), and VA plays an important role in processing visual information (Uchiyama et al., 2003). Understanding the cooperation mechanism of these brain areas during driving can help explain how the cognitive workload influences the brain network. In this study, EC was measured using CGC, which is deemed suitable for multivariate time series data and requires no prior knowledge of the directions of connectivity among different brain areas (Gao et al., 2011). This study aimed to achieve the following goals: (1) to investigate the activation status between actual driving and the resting state; and (2) to assess the EC among different brain areas and examine how cognitive workload affects such connectivity.

## Materials and Methods

### Subjects

Twelve young male subjects were recruited from the university for this study (age: 24.5 ± 2.8 years, BMI: 23.6 ± 2.3). The subjects were not under psychotropic medication (e.g., stimulants, anti-depressants and anxiolytics) and had no history of neurological injury or disease, seizure disorder, or psychiatric diagnosis based on an interview. All subjects were right-handed according to the Edinburgh Handedness Inventory (Oldfield, 1971) and were clear of the following characteristics: hypertension, neurological or psychiatric diseases, smoking or drinking habits and abnormal heart, lung, and kidney functions. All subjects were paid for their participation. The experimental procedure was approved by the Human Ethics Committee of the National Research Center for Rehabilitation Technical Aids and was conducted in accordance with the ethical standards specified by the Helsinki Declaration of 1975 (revised in 2008). Written informed consent was obtained from all subjects before study enrollment.

### Experimental Procedure and Measurements

**Figure 1:**
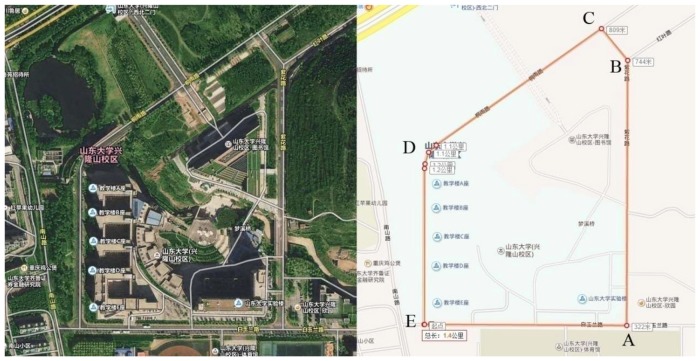
Experiment location. The driving route is cycling as A-B-C-D-E-A.

This experiment was performed on an annular route in the Xinglong Mountain Campus of Shandong University, Jinan, China (Figure 1). This experimental route is approximately 1400 m long and covers five corners (A-E), where the A-B-C section is slightly downhill, the C-D-E section is flat, and the E-A section is slightly uphill.

**Figure 2:**
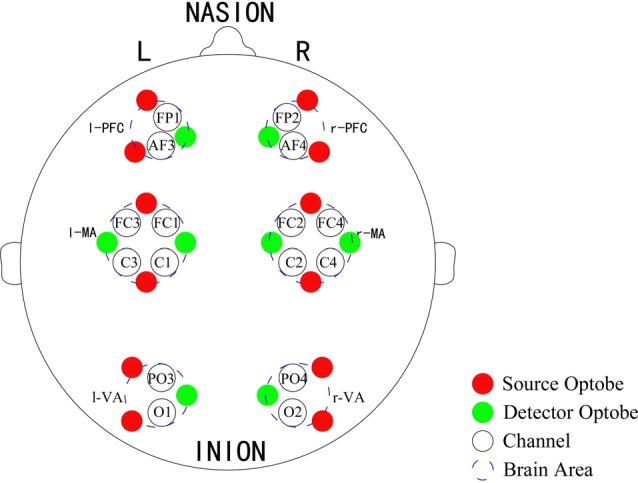
Optode placement of 16 channels. Red dots denote the source optodes, green dots denote the detector optodes, and blue dashed circles represent the corresponding brain areas. The measurement channels (black circles) in the prefrontal cortex (PFC), motor-related areas (MA) and vision-related areas (VA) are in accordance with the international 10/10 system.

A Toyota Yaris-2011 subcompact car and a Toyota E’Z-2011 FUV were used for this experiment. The former was used as the test car and the latter was used as the leading car. For the test car, a 2000W inverter was used to convert the 12V DC of the vehicle battery to 220V AC for the power supply of the NIRS instrument and laptop. A multi-channel tissue oxygenation monitor (NirSan Danyang Huichuang Medical Equipment Co. Ltd.) was utilized to record the delta [HbO_2_] of the subjects. The optodes were positioned over left PFC (l-PFC: FP1 and AF3), right PFC (r-PFC: FP2 and AF4), left MA (l-MA: FC1, FC3, C1 and C3), right MA (r-MA: FC2, FC4, C2 and C4), left VA (l-VA: O1 and PO3), and right VA (r-VA: O2 and PO4) with 16 channels in accordance with the International 10/10 System (Table 1, Figure 2). The sampling rate was set to 10 Hz.

**Table 1:** Distribution of the channels in the six brain areas.

Brain regions	Channels
Right prefrontal cortex (r-PFC)	FP2, AF4
Left prefrontal cortex (l-PFC)	FP1, AF3
Right motor area (r-MA)	FC2, FC4, C2, C4
Left motor area (l-MA)	FC1, FC3, C1, C3
Right visual area (r-VA)	PO4, O2
Left visual area (l-VA)	PO3, O1

The experiment was divided into three sessions, namely, resting, task_1 (simple-driving) and task_2 (car-following) states. Each session lasted 5 min following a 5-min rest after each session. The subjects were instructed to familiarize themselves with the protocol for performing the tasks prior to the experiment. In the resting state, the subjects were instructed to remain still and relaxed with their eyes open, hands on the steering wheel and feet on the pedals while remaining seated on the driver’s seat. In the task_1 state, the subjects were required to drive in the right lane within a speed of around 25 km/h. In the task_2 state, the driver in the leading car was asked to drive at a constant speed of 25 km/h. The subjects in the test car were asked to drive at a safe distance from the leading car and maintain the distance as far as possible. In the resting state, the engine was turned on as air conditioning was needed to maintain a comfortable temperature in the car. After each session, subjects were asked to complete the NASA Task Load Index (NASA-TLX), whose rating range was from 0 (no cognitive workload) to 100 (heavy cognitive workload), to rate the subjective cognitive workload (Hart and Staveland, 1988). Two persons (the subject and the recorder) were in the car during the entire experiment session.

### Data Processing

#### Pre-Processing

The data pre-processing and wavelet-based coherence analysis methods have been described in our previous studies (Bu et al., 2016; Tan et al., 2016; Wang B. et al., 2016). In the present study, first, a five-order Butterworth band-pass filter was used: the frequency elements below 0.021 Hz, which were mainly physiological noises of endothelial activities, and the elements above 0.145 Hz, which mainly reflected the respiration and cardiac activities, were removed (Shiogai et al., 2010). Then the moving average method was used to remove the outlier terms in the delta [HbO_2_] signals.

#### Wavelet Amplitude

Wavelet transform is a common method for transforming time series data from the time domain to the time-frequency domain with appropriate time and frequency resolution. The Morlet wavelet, which is a Gaussian function modulated by a sine wave with basic frequency, was used as a mother wavelet to detect the frequency content. The wavelet amplitude (WA) was the time-averaged wavelet transform amplitude of delta [HbO_2_], which can also indicate the frequency properties over the time domain.

#### Conditional Granger Causal Analysis

The following procedure was mainly accomplished with the GCCA toolbox (Seth, 2010). The basic idea of GC is that for time series X and Y, if knowing the past information of X helps to predict the future Y, X is assumed to “cause” Y, which is shown as *F*_*X*→*Y*_. CGC is an extension of GC in multivariate autoregressive models by “conditioning out” the influences of the other time series (Barnett and Seth, 2014; Wang L. et al., 2016). For time series X and Y chosen as the source and the target, respectively, all the other time series are composed of Z values, shown as *F*_*X*→*Y*|*Z*_. Each step of this method is described in detail as follows.

The data was first detrended and demeaned; then, the Augmented-Dickey-Fuller test and the Kwiatkowski-Phillips-Schmidt-Shin test were performed to check the covariance stationarity (Chatfield and Fuller, 1977; Kwiatkowski et al., 1990). The model order was identified using the Akaike information criterion and Bayesian information criterion (Akaike, 1974; Schwarz, 1978).

Second, a multivariate autoregression model was established to calculate the CGC of the data. The GC values of each connection between two channels were calculated under a Bonferroni-corrected significance threshold of *p* = 0.01 (Seth, 2010).

Three tests were performed to test the validity of the proposed model: the Durbin-Watson test checked whether the residuals were serially uncorrelated (Durbin and Watson, 1950), the consistency test was applied to assess the portion of data captured by this model (Ding et al., 2000), and a third test was performed as a supplementary test for consistency to analyze the adjusted sum square error (Seth, 2010).

The existence and the strength of a connection must be ensured to construct a connectivity graph. On the one hand, for a single subject, the existence of one connection between two channels was determined by the GC values under a Bonferroni-corrected significance threshold of *p* = 0.01. At group level, if the connections between two channels exist over 75% of subjects (Park and Friston, 2013), it was considered to have a connection between these two channels. In the connectivity graph, if the connections between two brain areas exist over 75% of connections between any two channels from these two brain areas, a connection was considered to exist a connection between these two brain areas in this study. On the other hand, the strength of each connection is defined as the average GC values between two brain areas. The GC values of the subjects in the three states were then averaged to establish three matrices, whose elements denoted the average GC values from one brain area to another.

#### Causal Flow

One additional index, causal flow, was applied after establishing an EC graph. The causal flow of a brain area, which is denoted as node *i* in a connectivity graph, is defined as the difference between its in-degree and its out-degree (Seth, 2009). A brain area with a positive causal flow exerts a strong causal influence on a causal network and can be referred to as a causal source. By contrast, a node with negative causal flow can be referred to as a causal target.

#### Statistical Analysis

The Kolmogorov-Smirnov test and the Levene test were performed in this study to ensure that the obtained values met the assumption required by the analysis of variance (ANOVA) analysis. One-way repeated ANOVA was performed to assess the main differences in the NASA-TLX scores, WA, matrices of average GC values, and causal flows among the resting, task_1 and task_2 states. Then the *post hoc* test was performed. The statistical significance was set to *p* < 0.05 with Bonferroni-correction for pair-wise comparisons.

## Results

### Cognitive Workload

**Figure 3:**
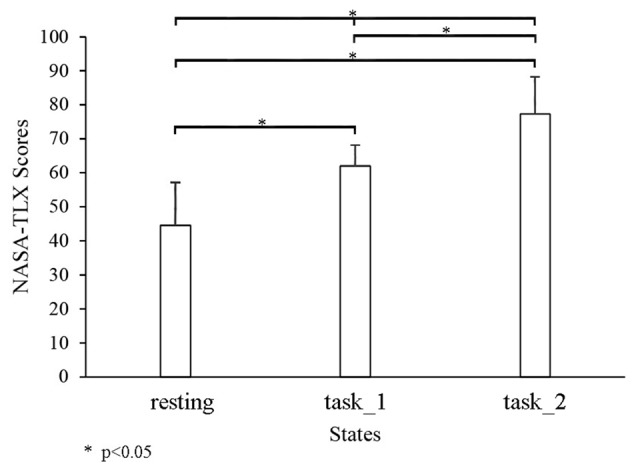
Comparison of the NASA-task load index (TLX) among three states. “*” Indicates a statistically significant change.

Figure 3 shows the comparison of the cognitive workload among three states through NASA-TLX. The NASA-TLX scores significantly increased as the states changed from the resting state to the task_2 state. Significant changes were observed among the three states (resting-task_1-task_2: *p* < 0.05, *F*_(2,33)_ = 45.3305; resting-task_1: *p* < 0.05, *F*_(1,22)_ = 55.5834; resting-task_2: *p* < 0.05, *F*_(1,22)_ = 92.2196; and task_1-task_2: *p* = 0.0428, *F*_(1,22)_ = 4.6235).

### Wavelet Amplitude

**Figure 4:**
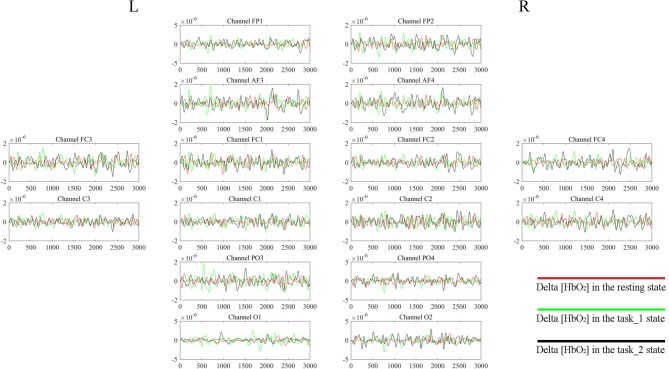
Delta [HbO_2_] signals of one subject. The distribution of the channels is the same as that in Table 1 and Figure 2. For each channel, the horizontal *X*-axis shows the time and the vertical *Y-axis* shows the delta [HbO_2_] signals. The signals of different colors represent different delta [HbO_2_] signals in different states.

**Figure 5:**
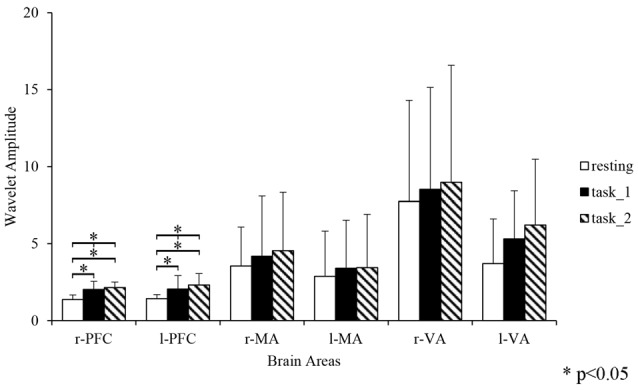
Comparison of the WA in the six brain areas among the three states. “*” Indicates a statistically significant change. Abbreviations: r-PFC, right prefrontal cortex; l-PFC, left prefrontal cortex; r-MA, right motor-related areas; l-MA, right motor-related areas; r-VA, right vision-related areas; l-VA, left vision-related areas; WA, wavelet amplitude.

Figure 4 gives an example of the delta [HbO_2_] signals of one subject after pre-processing. As shown in the picture, for most channels, especially the channels in VA, the fluctuations of delta [HbO_2_] signals in the task_2 state were larger than in the other two states. Figure 5 shows a comparison of the WA of the subjects in six brain areas among the three states. The WA of right MA and VA was higher than that of the left part of the brain in MA and VA. The WA increased with an increase in the cognitive workload. According to the ANOVA test results, statistically significant differences were observed in bilateral PFC among the three states (resting-task_1-task_2; r-PFC: *p* = 0.0071, *F*_(2,33)_ = 5.7682; l-PFC: *p* = 0.0298, *F*_(2,33)_ = 3.9172), resting-task_1 state (r-PFC: *p* = 0.0072, *F*_(1,22)_ = 8.7867; l-PFC: *p* = 0.0157, *F*_(1,22)_ = 6.8510), and resting-task_2 state (r-PFC: *p* = 0.0028, *F*_(1,22)_ = 11.3614 ; l-PFC: *p* = 0.0102, *F*_(1,22)_ = 7.8985).

### EC Graphs

**Figure 6:**
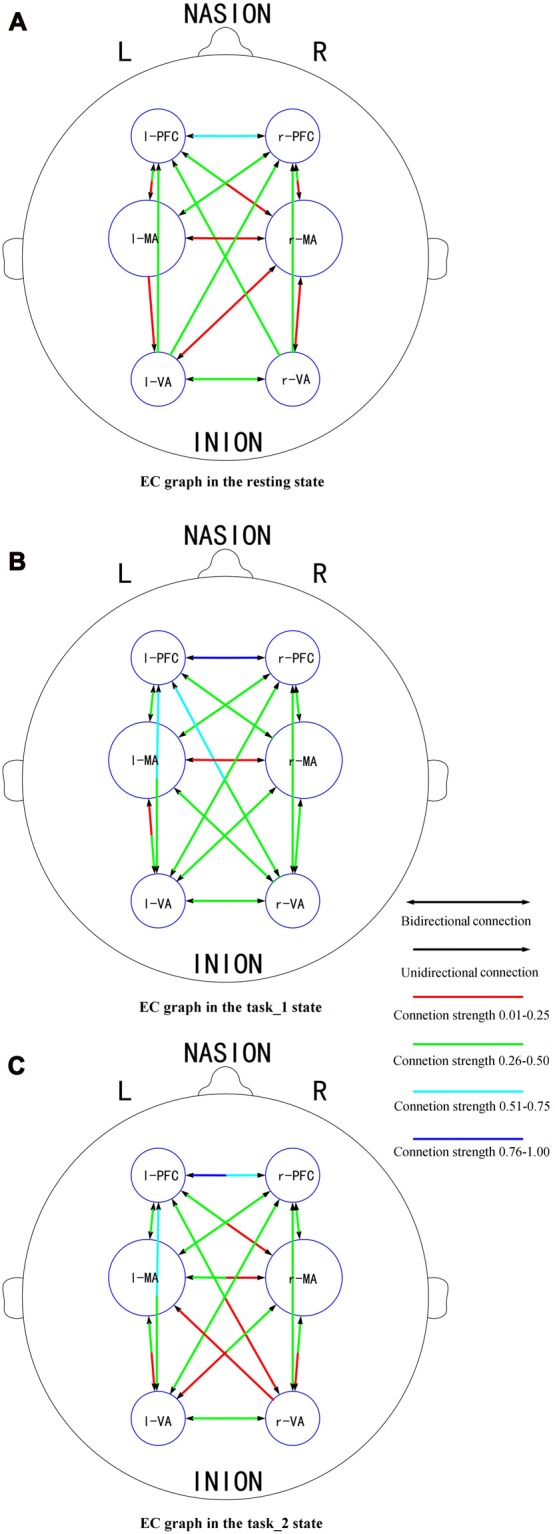
EC graphs of six brain areas in the **(A)** (resting), **(B)** (task_1) and **(C)** (task_2) states, respectively. The connection with double-headed arrows denotes the connection in EC is bidirectional. The connection with single head arrow denotes the connection in EC is unidirectional. Different colors represent different connection strength. The colder color represents the stronger connection strength. Abbreviations: r-PFC, right prefrontal cortex; l-PFC, left prefrontal cortex; r-MA, right motor-related areas; l-MA, right motor-related areas; r-VA, right vision-related areas; l-VA, left vision-related areas; EC, effective connectivity.

**Figure 7:**
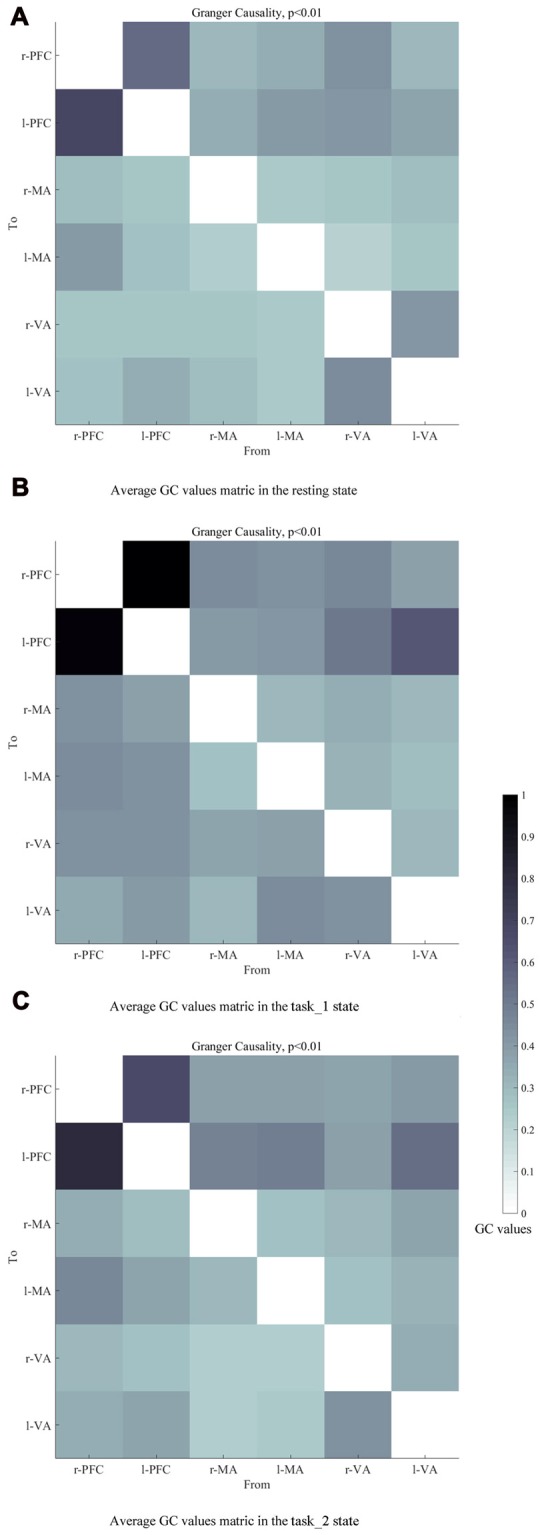
Average GC value matrices of six brain areas in the **(A)** (resting), **(B)** (task_1) and **(C)** (task_2) states, respectively. Each element in the matrix denotes the average GC values across all subjects. The causal line is from bottom row to the left column. The darker color of the block represents the higher GC values of the connection. Abbreviations: r-PFC, right prefrontal cortex; l-PFC, left prefrontal cortex; r-MA, right motor-related areas; l-MA, right motor-related areas; r-VA, right vision-related areas; l-VA, left vision-related areas; GC, granger causality.

Figures 6A–C presents the GC connectivity graphs of six brain areas in the three states, respectively. Figures 7A–C presents the average GC value matrices of six brain areas in the three states, respectively. Table 2 compares the connection strength among the three states.

**Table 2:** Comparison in the connection strength of the subjects among three states (A; resting-task_1-task_2), (B; resting-task_1), (C; resting- task_2) and (D; task_1-task_2).

	(A) resting-task_1-task_2					
From / To	r-PFC	l-PFC	r-MA	l-MA	r-VA	l-VA
r-PFC		(*)			(*)	
l-PFC					(*)	
r-MA						
l-MA						
r-VA						
l-VA						
	(B) resting-task_1					
From / To	r-PFC	l-PFC	r-MA	l-MA	r-VA	l-VA
r-PFC		(*) +				
l-PFC						
r-MA						
l-MA					appear	appear
r-VA	appear	appear		appear		
l-VA	appear	appear				
	(C) resting-task_2					
From / To	r-PFC	l-PFC	r-MA	l-MA	r-VA	l-VA
r-PFC						
l-PFC						
r-MA						
l-MA					appear	appear
r-VA	appear	appear	lost	appear		
l-VA	appear	appear				
	(D) task_1-task_2					
From / To	r-PFC	l-PFC	r-MA	l-MA	r-VA	l-VA
r-PFC		(*) −			(*) −	
l-PFC					(*) −	
r-MA						
l-MA						
r-VA			lost			
l-VA						

“Appear” denotes that the connection does not exist in the former state but appears in the following state and “lost” indicates* vice versa*. “*” Denotes that the average Granger causality (GC) values changes significantly among the states in comparison. “+” Indicates that the change is a significant increase. “−” Denotes that the change is a significant decrease. Abbreviations: r-PFC, right prefrontal cortex; l-PFC, left prefrontal cortex; r-MA, right motor-related areas; l-MA, right motor-related areas; r-VA, right vision-related areas; l-VA, left vision-related areas.

There were more connections among brain areas in the task states than in the resting state. The connections from bilateral PFC to bilateral VA, from bilateral VA to l-MA, and from l-MA to r-VA “appeared” from the resting state to the task_1 state. The connection from r-MA to r-VA was “lost” from the task_1 state to the task_2 state. For the connection strength, among the three states (resting-task_1-task_2), the connection strength of connections from l-PFC to r-PFC (*p* = 0.0184, *F*_(2,33)_ = 4.5173), from r-VA to r-PFC (*p* = 0.0375, *F*_(2,33)_ = 3.6327), and l-PFC (*p* = 0.0423, *F*_(2,33)_ = 3.4863) showed significant difference. From the resting state to the task_1 state, the connection strength of the connection from l-PFC to r-PFC increased significantly (*p* = 0.0158, *F*_(1,22)_ = 6.8365). From the task_1 state to the task_2 state, the connection strength of the connection from l-PFC to r-PFC (*p* = 0.0137, *F*_(1,22)_ = 7.1812), from r-VA to r-PFC (*p* = 0.0152, *F*_(1,22)_ = 6.9311) and l-PFC (*p* = 0.0109, *F*_(1,22)_ = 7.7326) decreased significantly.

### Causal Flow

**Figure 8:**
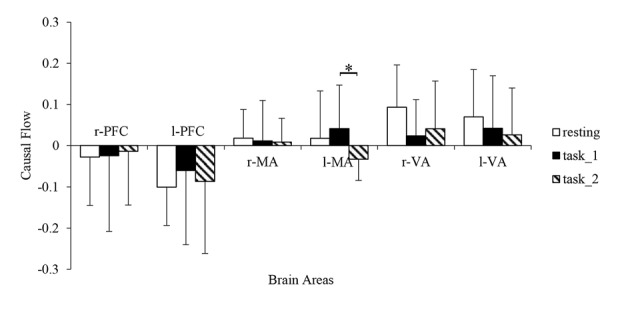
Comparison of the causal flows in the six brain areas among the three states. “*” Indicates a statistically significant change. Abbreviations: r-PFC, right prefrontal cortex; l-PFC, left prefrontal cortex; r-MA, right motor-related areas; l-MA, right motor-related areas; r-VA, right vision-related areas; l-VA, left vision-related areas.

The granger causal flows are shown in Figure 8. In the resting state, the bilateral PFC can be considered as causal targets while bilateral MA and VA can be considered as causal sources. In the task_1 state, the status remained the same. In the task_2 state, the l-MA became a causal target and the in-out degree of l-MA decreased significantly from the task_1 state to the task_2 state (*p* = 0.0114, *F*_(1,22)_ = 7.6337).

## Discussion

The changes in the EC of the brain network among PFC, MA and VA in the resting, simple-driving and car-following states were assessed in this study using CGC. Several interesting findings were obtained: (1) the WA changes showed the same trend as that of the cognitive workload from the resting state to the task states. The WA of left MA and VA was higher than that of the right brain areas, and the WA of the bilateral PFC changed statistically significantly along with the change in the cognitive workload; (2) the connection strength of EC increased in the task_1 state as compared with that in the resting state, whereas it decreased in the task_2 state as compared with that in the task_1 state; and (3) the bilateral PFC were causal targets, and the bilateral VA and MA were causal sources except for l-MA became a causal target in the task_2 state. These findings suggest that the EC of the brain network can be strengthened by a cognitive workload, and can also be weakened by a superfluous cognitive workload such as driving with a car-following task.

The typical hemodynamic response to brain activation is the basis for NIRS measurement. When a specific brain area is activated, neural metabolism is supported through a localized vascular response that causes an influx of oxygen-rich blood to the active area and the surrounding tissue. This phenomenon leads to an increase in [HbO_2_] and a decrease in [HbR] in the active brain area (Matthews et al., 2008). The functional hyperemia mechanism adjusts the distribution of cerebral blood flow on the basis of the functional activities of different brain regions (Iadecola, 2004). Therefore, when the activity level of the cerebral cortex region increases or decreases, the blood flow in this area changes accordingly, and this change can be reflected by the fNIRS signals ([HbO_2_]).

It was considered that the cerebral NIRS signals originated from neurovascular coupling and systemic activity components (Holper et al., 2014). The sympathetic nervous system and vascular myogenic responses could play a part in neurovascular coupling (Schroeter et al., 2004; Hamner et al., 2010). The signals originate from the intrinsic myogenic activity of smooth muscle cells in resistance vessels and are regulated by the neurogenic activity in the vessel wall (Shiogai et al., 2010). A change in the WA reflects a change in the brain hemodynamic activity level in the neurovascular systemic activity components among different cognitive driving workloads.

Previous studies have highlighted the important role that PFC plays in executive attentional control, eye movement generation, cognitive control and other abstract reasoning functions (Miller and Cohen, 2001). MA have been correlated with the planning, control, and execution of body movements (Zhang et al., 2010). VA, as the visual processing center, receive visual information stemming from the eyes and elicits visual stimuli as object recognition and visuospatial guidance to the other cortex areas (Uchiyama et al., 2003). The WA of bilateral PFC changed significantly with the change of the cognitive workload indicated by the NASA-TLX scores. The increases in the WA were statistically significant from the resting state to the task states, while the increase between the task_1 and the task_2 states did not exhibit such change. This finding may be attributed to the fact that PFC was more sensitive to the change of cognitive work state. The car-following task needed more visuomotor control than the simple-driving task, which was related more to VA than to PFC and MA (Uchiyama et al., 2008). The WA of VA was clearly higher than that of PFC and MA. The significant right lateralization of the WA in VA was possibly attributed to the fact that the left hemisphere directs attention to the right space, but the right hemisphere directs attention to both spaces (Heilman and Van Den Abell, 1980; Kashiwagi et al., 1990). The right lateralization of the WA in MA was probably due to the fact that all subjects were right-handed, and therefore manipulation with their left hand was considerably harder than that with their right hand. Furthermore, according to Chinese laws, the vehicle is driven in the right lane, thus the visual stimuli come mainly from the left side of the view. This may explain the significant right lateralization of the WA in VA.

The directions and the connection strength of the connections of EC can reflect the influence of one brain region on another (Park and Friston, 2013). Some studies have revealed that movement impulses generated by the supplementary motor and premotor cortexes are induced by two sources: one is the occipital-parietal lobes of the posterior attention system, which correlates with the visual-spatial orientation and integration functions; and the other is PFC from the anterior attention system, which serves a relatively higher-level attention function such as executive attentional control in more complex cognitive tasks associated with problem-solving and decision-making (Posner, 2012). For the visual motion information, it is considered to be generated by VA, and then follows two main streams: one follows the dorsal stream to the parietal lobe, and the other follows the ventral stream to the temporal lobe, then the two streams eventually converge in PFC. PFC generates the stimuli for eye movements to obtain visual information (Schiller and Chou, 1998; Rizzolatti and Matelli, 2003).

In this article, the existence of a group-level effective connection between two areas was ensured by a threshold of 75% of subjects. As shown on the constructed EC graphs in this study, the brain activity information transitions among PFC, MA and VA were well connected in the task states. Bilateral PFC to bilateral VA connections “appeared” as the need for visual information increased from the resting state to the task_1 state. This is the same reason that the connection “appeared” from l-MA to r-VA. The connections from bilateral VA to l-MA “appeared” as driving manipulations were mainly implemented by the right hand under the guidance of visual information. During the task_2 state, the connection from r-MA to r-VA became “lost”, which implies that for the car-following task, the feedback neural information transition from r-MA to r-VA decreased, and the influence exertion from r-VA to l-MA relatively increased.

The connection strength from r-VA to bilateral PFC decreased significantly from the task_1 state to the task_2 state. When performing the car-following task, the subjects would concentrate more on the view in front of the car and less on the view on the left and right sides, resulting in a decrease in the influence strength from r-VA to bilateral PFC. Furthermore, the connection strength from l-PFC to r-PFC increased from the resting state to the task_1 state, whereas it decreased during the task_2 state compared with that in the task_1 state. The above mentioned increase implies that driving as an intense cognitive behavior can help strengthen the connection between the bilateral PFC for attention control, planning, decision making and other functions (Yoshino et al., 2013b). The car-following task imposed a larger cognitive workload on subjects than the simple-driving task. This increase in the cognitive level led to a decrease in the coordination of bilateral PFC for attention maintenance and cognitive control.

The positive in-out degree implies that brain area is a causal source in the EC, and the negative degree implies that the brain area served as a causal target. From the resting state to the task states, the in-out degree of PFC exhibited an increasing trend. This trend implies that with an increase in the cognitive workload, the activity of PFC for cognitive function also increases. Additionally, the increase corresponded with the decreasing connection strength from l-PFC to r-PFC. Furthermore, left dorsolateral PFC is necessary for manipulating information in the working memory and right dorsolateral PFC is critical for manipulating information in a broader range of reasoning contexts (Barbey et al., 2013). The in-out degree asymmetry between r-PFC and l-PFC was consistent with the different levels of need for spatial reasoning and visuomotor control for different cognitive workload behaviors. As VA execute the function of receiving visual information and eliciting visual stimuli to other areas (Uchiyama et al., 2003), they always presented as causal sources, and the in-out degree decrease of r-VA matched the decrease in the connection strength from r-VA to bilateral PFC. From the task_1 state to the task_2 state, the in-out degree of l-MA, which controlled the movement function of the contralateral part of the body, exhibited a statistically significant decrease. In the task_2 state, the right foot performed more manipulations for the throttle and brake than in the task_1 state, and l-MA was influenced considerably by the visual stimuli.

## Conclusion

In this study, the EC was calculated and analyzed using CGC for actual driving situations. The hemodynamic activity level of brain areas showed an increasing trend with the increase in the cognitive workload, particularly in bilateral PFC. The connection strength of EC was enhanced from the resting state to the simple-driving state, but it deteriorated during the car-following task. The PFC in EC appeared as the causal targets while MA and VA appeared as the causal sources. However, with a subtask of car-following, l-MA turned into causal targets. These findings indicate that the hemodynamic activity level of brain increases linearly with an increase in the cognitive workload. Furthermore, the EC of the brain network can be strengthened by a cognitive workload, and can also be weakened by a superfluous cognitive workload such as driving with a car-following task. This study proposes a method for evaluating the effects of the cognitive workload of drivers.

## Limitations of The Study

One limitation of this study was the interference of Mayer waves (0.08–0.1 Hz). Mayer waves are oscillations of arterial pressure occurring spontaneously in conscious subjects at a frequency lower than that of respiration and are tightly coupled with the synchronized oscillations of the efferent sympathetic nervous activity (Julien, 2006). As physiological noise induced by the global systemic physiological processes in fNIRS measurement, Mayer waves may affect the EC. Although the task-related changes in connectivity mainly reflects the synchronization of neurovascular coupling, the interference of Mayer waves should be taken into account in a future study. In addition, the number of subjects included in the present work was relatively limited in light of the current standards for neuroimaging experiments (Friston, 2012). Third, future research will focus on activation level in the standard space using NIRS-SPM (Ye et al., 2009).

## Author Contributions

ZLi and QY conceived and designed the study. ZLiu did the experiment, analyzed the data and drafted the manuscript. GX did the experiment and analyzed the data. CH performed the statistical analysis. QT designed the driving task. MZ contributed to the physiological interpretation of the results and edited the manuscript.

## Conflict of Interest Statement

The authors declare that the research was conducted in the absence of any commercial or financial relationships that could be construed as a potential conflict of interest.
